# Survival Analysis of Faculty Retention and Promotion in the Social Sciences by Gender

**DOI:** 10.1371/journal.pone.0143093

**Published:** 2015-11-18

**Authors:** Janet M. Box-Steffensmeier, Raphael C. Cunha, Roumen A. Varbanov, Yee Shwen Hoh, Margaret L. Knisley, Mary Alice Holmes

**Affiliations:** 1 Department of Political Science, The Ohio State University, Columbus, Ohio, United States of America; 2 Department of Statistics, Florida State University, Tallahassee, Florida, United States of America; 3 School of Medicine, Case Western Reserve University, Cleveland, Ohio, United States of America; Université de Montréal, CANADA

## Abstract

**Background:**

Recruitment and retention of talent is central to the research performance of universities. Existing research shows that, while men are more likely than women to be promoted at the different stages of the academic career, no such difference is found when it comes to faculty retention rates. Current research on faculty retention, however, focuses on careers in science, technology, engineering, and mathematics (STEM). We extend this line of inquiry to the social sciences.

**Methods:**

We follow 2,218 tenure-track assistant professors hired since 1990 in seven social science disciplines at nineteen U.S. universities from time of hire to time of departure. We also track their time to promotion to associate and full professor. Using survival analysis, we examine gender differences in time to departure and time to promotion. Our methods account for censoring and unobserved heterogeneity, as well as effect heterogeneity across disciplines and cohorts.

**Results:**

We find no statistically significant differences between genders in faculty retention. However, we do find that men are more likely to be granted tenure than women. When it comes to promotion to full professor, the results are less conclusive, as the effect of gender is sensitive to model specification.

**Conclusions:**

The results corroborate previous findings about gender patterns in faculty retention and promotion. They suggest that advances have been made when it comes to gender equality in retention and promotion, but important differences still persist.

## Introduction

The issue of gender equality in the workplace has been a public concern for decades and is pertinent in various fields and work environments. In academia and the sciences, career advancement has been typically described as a leaky pipeline where men and women “leak” at different rates as they move up the professional ladder. In many fields, men and women tend to enter graduate programs in similar numbers; however, throughout the different stages of the academic career women tend to drop out at higher rates and end up being substantially under-represented at higher ranks [1].

For research institutions, this could mean an inefficient use of intellectual and creative resources. Concern with faculty retention and promotion is typically based on the fact that the loss of a faculty member costs universities a great deal, as research start-up costs can be substantial in some fields and premature leaves can disrupt teaching and mentoring, which imposes additional costs to these institutions [2].

Despite the common metaphor of the leaky pipeline, universities and advising institutions, such as the National Academies, have worked hard to devise policies that support women and men equally in their academic careers [3, 4, 5] and to improve gender diversity in faculty recruitment. The National Science Foundation’s ADVANCE program [6], for example, aimed at “increasing the participation and advancement of women in academic science and engineering careers,” has led to transformational programs, such as the University of Michigan’s STRIDE program [7], which offers excellent examples of best practices for de-biasing faculty search processes, Washington State University’s ADVANCE Program on mentoring [8], Texas A&M’s ADVANCE Program on retention and promotion [9], and the University of California at Davis’ ADVANCE Program for creating a level playing field for success [10]. Similarly, Ohio State University’s Kirwan Center offers resources on curbing implicit gender bias [11], and the Visions in Methodology (VIM) Conferences support women in the field of political methodology by providing a forum to share scholarly work and to connect women in a field where they are under-represented [12]. And yet, despite a number of good policies, many still share the perception that progress has not happened at the desired pace [13].

### Sources of Gender Differences in Retention and Promotion

Our expectations about gender differences in time to promotion and time to departure are based on previous research on career advancement in science. Here we discuss our expectations regarding gender-based patterns in the risk of promotion to the associate and full professor rank and the risk of departure from one’s institution of employment.

Gender inequality manifests itself at various career stages. The first filter in career advancement stands between graduation from a Ph.D. program and admission to a tenure-track faculty position. Existing studies show that women and men in various fields tend to enter graduate programs in similar numbers; however, men are more likely to become academic scientists upon graduation [14, 15]. It has also been shown that having young children negatively impacts women’s chances of moving onto the tenure track but has no effect on men’s chances [16, 15]. Moreover, while women receive about half of the graduate and post-doctoral fellowships from the National Institutes of Health (NIH), this parity has not translated into gender parity in grant funding at the faculty level [17]. Success rates for grant applications are similar for men and women; rather, the disparity in grant funding seems to come from low female representation among faculty. As one scholar put it, “nearly the entire leak in the pipeline occurs at the transition between the postdoctoral period and junior faculty” [17].

Gender-based patterns also appear at the pre-tenure stage and the tenure review process. When it comes to the tenure decision, research productivity is widely considered the most decisive factor [18, 19, 20, 21]. However, even controlling for productivity, studies have found women to be less likely to be granted tenure or to take more time to do so [15, 22, 23, 24, 25]. A study by Junn found that women and racial minorities were less likely to be awarded tenure at the University of Southern California [26, 27], and another recent study reported that, taking into account research productivity and the prestige of the research outlet, men were more likely to be awarded tenure than women in the fields of computer science, English, and sociology [28, 29]. In contrast, an analysis of time to tenure for science, biomedical, and engineering faculty in Spanish universities found that “the role of sex is minor” [30].

Several explanations have been proposed for commonly observed differences in academic productivity between men and women. First, female scientists are less likely to hold the positions and have access to the facilitating resources that are conducive to higher rates of publication performance [31]. Similarly, lower publication rates of female faculty are correlated with the level of resource-intensity of research in different fields, which “may be explained by the lower level of institutional support historically received by females” [24]. In addition, differences in promotion rates between female and male academic personnel typically cannot be explained by gender differences in working hours, but may be explained by differences in years of service and external mobility [25]. Women have also been found to be more likely than men to devote time to teaching and advising and teach in fields unlike the ones in which they were trained [32]. And, lastly, women tend to spend more time on household and childcare responsibilities [33, 34].

In addition, factors that do not directly bear on differential productivity rates may also help create differences in rates of tenure promotion. Citation patterns in the field of International Relations shows that women are systematically cited less than men even after controlling for factors such as quality of publication, substantive focus, theoretical perspective, methodology, tenure status, and institutional affiliation [35]. Moreover, gender differences in promotion rates are associated with differences in research focus (traditional subjects vs. newer subfields), methodologies (quantitative vs. qualitative), as well as different perceptions of official and unwritten expectations for research [36].

Another factor that may influence tenure appointments for women is gender bias in student evaluations. Students tend to devalue the credentials of female professors, ascribing the term ‘professor’ to male academics, even if they are only graduate students, while female professors are described as ‘teachers’ [37], and students who receive a low grade tend to be more critical of the professor if the instructor is female rather than male [38]. Laube et al. suggest that this line of research is indicative of a structural problem with academia which puts women at a disadvantage [39].

Such a combination of factors leads us to expect gender differences in rates of tenure promotion. Moreover, many of those factors should also lead to differential expectations when it comes to promotion to full professor. Different levels of institutional support, gendered citation patterns, and imbalances in the division of domestic labor should also lead to differences in research productivity after tenure and to lower chances of promotion to full professor for women. Indeed, studies of promotion from associate to full professor have found that women are systematically less likely to achieve said promotion than men [15, 22].

Given the above-discussed institutional factors that impinge on gender differences in faculty productivity and impact, and considering the prominent role of promotion in the retention of talent, we should also expect to observe differences across genders in faculty retention. Disadvantages in access to material resources, institutional support, citations, the division of domestic chores, among others, which translate into a disadvantage in evaluations for promotions, should also translate into lower retention rates for female faculty. As they face higher costs for advancing in their career, we should expect women to drop out at higher rates.

Previous research provides mixed evidence on the existence of gender imbalances in faculty retention. A study by Kaminski and Geisler of faculty retention in science and engineering in fourteen U.S. universities found no systematic differences in retention rates by gender in the nine STEM fields studied, with the exception of mathematics [2]. In turn, previous studies have found that, while men’s most cited motive for leaving a research institution is financial, women tend to leave universities before they reach tenure because of “interpersonal and family reasons” [40]. This suggests that factors that explain differences in promotion rates should also cause retention rates to differ by gender.

Much of the attention over issues of gender imbalances in academia has focused on STEM fields, and the social sciences have seen comparatively little research on the topic. We extend this line of inquiry into the social sciences to determine whether there are significant differences in time of employment and promotion rates of male and female faculty members in the social sciences. As such, this study is designed as an extension to Kaminski and Geisler [2].

## Data and Methods

### Data Collection

We track a total of 2,218 social science faculty members from seven disciplines in nineteen U.S. universities from their time of hire as assistant professor, starting in 1990, to the time they left the university or until 2009, whichever came first. The data were gathered through available college catalogs, online search engines, and by directly contacting universities. The specific disciplines that are examined within the social sciences are: anthropology, communication, economics, geography, political science, psychology, and sociology.

We include ten of the original fourteen universities from the Kaminski and Geisler study [2] and add nine more. The nineteen universities selected for analysis are: Boston University, Cornell University, George Washington University, Georgia Institute of Technology, Massachusetts Institute of Technology, Rensselaer Polytechnic Institute, University of Delaware, University of Massachusetts at Amherst, University of Rhode Island, Virginia Tech University, Northwestern University, Stanford University, The Ohio State University, University of California at Berkeley, University of Iowa, University of Michigan, University of Texas, University of Wisconsin, and Yale University.

All 2,218 faculty tracked fit the criteria of the study and had the available information that was needed. To obtain information for each university and faculty member, a variety of resources were used. Following a similar procedure to that used by Kaminski and Geisler [2] and Junn [27], our primary sources of data are college catalogs, which list faculty names and positions under each specific academic department. For the years 1990–1998, the college catalogs are available as microfiche files at the Ohio State University library. Most of the college catalogs for the more recent years are available online at the website www.collegesource.org, where searches can be entered for specific universities. In order to fill in incomplete data, we used multiple online sources with the aid of search engines. Missing information could include, for example, first or last names, year of departure, or year of promotion. When needed, university libraries and archives were directly contacted by phone. We also used the online service TaskRabbit (https://www.taskrabbit.com) to hire research assistants at other universities to collect some of the faculty data.

Each entering assistant professor was tracked from year to year by their last name and the year was noted for their entrance, promotion to associate and full professor, and their departure from the university. An assistant professor was first added to the data when their name initially appeared on the college catalog. Then, in the following years, the name was tracked to determine the years when he or she was promoted. If the name no longer appeared on the catalog, then the person was considered to have left the institution and the name was no longer tracked.

Following Kaminski and Geisler [2], we divide faculty members into 5 cohorts based on the year that they entered into employment at the university. Each cohort includes faculty members hired within a four-year span, starting with 1990–1993 and ending with 2006–2009. The cohorts vary in size and composition, which can be seen in Table 1.

**Table 1:** Gender distribution (cohorts 1–5).

*Cohort*	*Entry Point*	*Men*	*Women*	*Total*	*Proportion Women*
1	1990–1993	306	172	478	0.36
2	1994–1997	216	178	394	0.45
3	1998–2001	281	179	460	0.39
4	2002–2005	307	195	502	0.39
5	2006–2009	204	180	384	0.47
All		1,314	904	2,218	0.41

Finally, we checked for the possibility that faculty might have moved between universities during the period covered by the study. If the proportion of faculty who moved is substantial and there is a gender difference, then counting faculty twice could induce bias in the analysis. To avoid the problem, we checked the sample for duplicate names. 20 faculty moved between universities in our sample, which constitutes less than 1% of the total sample of 2,218. Of the 20 faculty who moved, 12 were men and 8 were women. We, therefore, have no reason to worry about any potential threats to inference arising from duplicate counting.

### Right-Censoring

The year of 2009 serves as the right-censoring year for the study. To understand the concept of censoring, we can think of time as moving from left to right on a horizontal axis. At the end of 2009, the year in which we stopped the data collection, many of the faculty members were still in activity on the tenure track at their institution, so we have no information on their actual time to departure. We thus treat these cases as right-censored.

It could be argued that the data are interval-censored. Since information on the time of leaving was collected at annual intervals, the exact time of leaving is unknown. Statistical methods for analyzing interval-censored data are available. However, since most faculty tend to leave towards the end of the academic year, we find that the assumption that the data are right-censored is a reasonable approximation. Moreover, no faculty that was already on the tenure track before 1990 was included in the study. Since we only included faculty members that were hired in or after 1990, the data are not left-censored.

### Statistical Analysis

We use survival analysis in order to determine if there are systematic differences across genders in time of employment and time to promotion within the social sciences during the period covered by the study. Our analysis only uses data for the faculty that started employment between 1990 and 2001 (cohorts 1–3). Faculty in the two most recent cohorts have not been in position long enough to provide useful information for the survival analysis. By restricting the analysis to this period, we also maintain consistency with Kaminski and Geisler [2] and ensure that the results are comparable.

We start by calculating nonparametric Kaplan-Meier survival curves by gender. Kaplan-Meier curves are useful for dealing with time-to-event differences across groups, especially when not all the subjects continue in the study (right-censoring), as is the case here. In terms of faculty retention, Kaplan-Meier curves show the cumulative probability of staying at one’s institution of employment at a given time. We use two nonparametric statistics to compare Kaplan-Meier survival curves across genders: a log-rank test and a Wilcoxon (Breslow-Gehan) test of the equality of survivor functions. To put it succinctly, at each event time in the data, the contribution to the test statistic is obtained as a weighted standardized sum of the difference between the observed and expected number of events in each group. Tests differ in the weights used. The log-rank test uses a weight of one at all event times and is most appropriate when the hazard functions are thought to be proportional across the groups [41]. The Wilcoxon (Breslow-Gehan) test is appropriate when hazard functions are thought to vary in nonproportional ways and when censoring follows a similar pattern across groups [42, 43].

We next correlate the data using a Cox Proportional Hazards model. The Cox PH model allows us to estimate differences in hazard (risk of departure) across genders without the need to make parametric assumptions about the functional form of the baseline hazard [44, 45]. We first estimate a Cox model where gender is the only included covariate. We code the gender indicator variable as one for male and zero for female.

Moreover, to address concerns about potential confounders, we also estimate a frailty model of time to departure for social science faculty. Frailty models are used to account for unobserved heterogeneity in time-to-event data. They assume that some observations are more prone to experiencing the event than others (i.e., they are more “frail”), but the source of that difference is unobserved. This modeling technique introduces into the hazard rate an additional random parameter that accounts for the random frailties [46, 47, 48]. We estimate a survival model with individual-specific frailties. A tractable model for the heterogeneity can be derived by making assumptions about the distribution of the frailty. It is typically assumed that the frailty follows a gamma probability distribution with mean one and unknown variance equal to a parameter *θ*. Incorporating unobserved heterogeneity into the survival model, therefore, involves estimating the additional random parameter *θ*. If the frailty variance, *θ*, is zero, then the model reduces to the standard proportional hazards model. A test of the frailty parameter can be conducted by means of a likelihood-ratio test [47]. We estimate parametric individual-frailty models using different specifications for the functional form of the baseline hazard and choose the specification with the better fit based on the standard Akaike information criterion (AIC).

To make sure that discipline-specific gender patterns are not obscured by a general analysis of the entire sample of faculty members included in this study, we also break down the survival analysis of faculty departure by social science discipline. We estimate a Cox model where the gender variable is interacted with discipline dummy variables and test for the equality of gender differences across disciplines using a standard Wald-type chi-square test for the equality of coefficients on the interaction terms. Additionally, we examine the possibility of heterogeneity in gender differences across different cohorts. We follow the same approach outlined above and interact the gender variable with cohort dummy variables.

A complementary way to examine faculty retention is to look at promotion rates. We begin by analyzing gender differences in promotion to tenure. We adopt the same design outlined above and estimate a Cox model for cohorts 1 to 3 in which the event of interest is now defined as promotion from assistant to associate professor. For each faculty, we record the duration from time of hire to time of promotion to associate level. Again, the data are right-censored, since not every faculty member that was observed was promoted to the associate rank by 2009, the year for which we stopped collecting the data. We estimate the same set of models described above, including an individual-frailty parametric survival model, a model with discipline-specific gender effects, and a model with cohort-specific gender effects.

In addition, we examine the time to promotion from associate to full professor by gender, conditional on the faculty member having been promoted to associate professor. In this case, faculty members enter the study when they are promoted to associate professor and the event of interest is defined as promotion to full professor. The analysis follows the same design as the one used in the analysis of promotion from assistant to associate professor. All computations were performed in Stata 11.

## Results

Table 2 gives the median time to departure, as well as the 25th and 75th percentiles, for cohorts 1 to 3 by gender. Half of all entering faculty have departed by year 9, and there is no statistically discernible difference between men and women. Estimates of the 95% confidence intervals for the median are the same for both groups. We also do not find significant differences at the 25th percentile. Survival times are unavailable at the 75th percentile; given the available data, we can estimate the 75th percentile to be at least 19 years. Table 3 provides sample sizes by discipline to help with the interpretation of discipline-specific results, as described below.

**Table 2:** Percentiles of time to exit the tenure track by gender (cohorts 1–3).

	*Number of subjects*	*25th*	*50th (median)*	*75th*
*All*	1332	5 (5, 6)	9 (8, 10)	≥ 19
*Men*	803	5 (4, 5)	9 (8, 10)	≥ 19
*Women*	529	5 (5, 5)	9 (8, 10)	≥ 19

Note: Values in parentheses are 95% confidence intervals.

**Table 3:** Sample size by discipline (cohorts 1–3).

*Discipline*	*Number of subjects*
Anthropology	116
Communication	176
Economics	272
Geology	66
Political Science	245
Psychology	277
Sociology	180
Total	1,332

**Fig 1:**
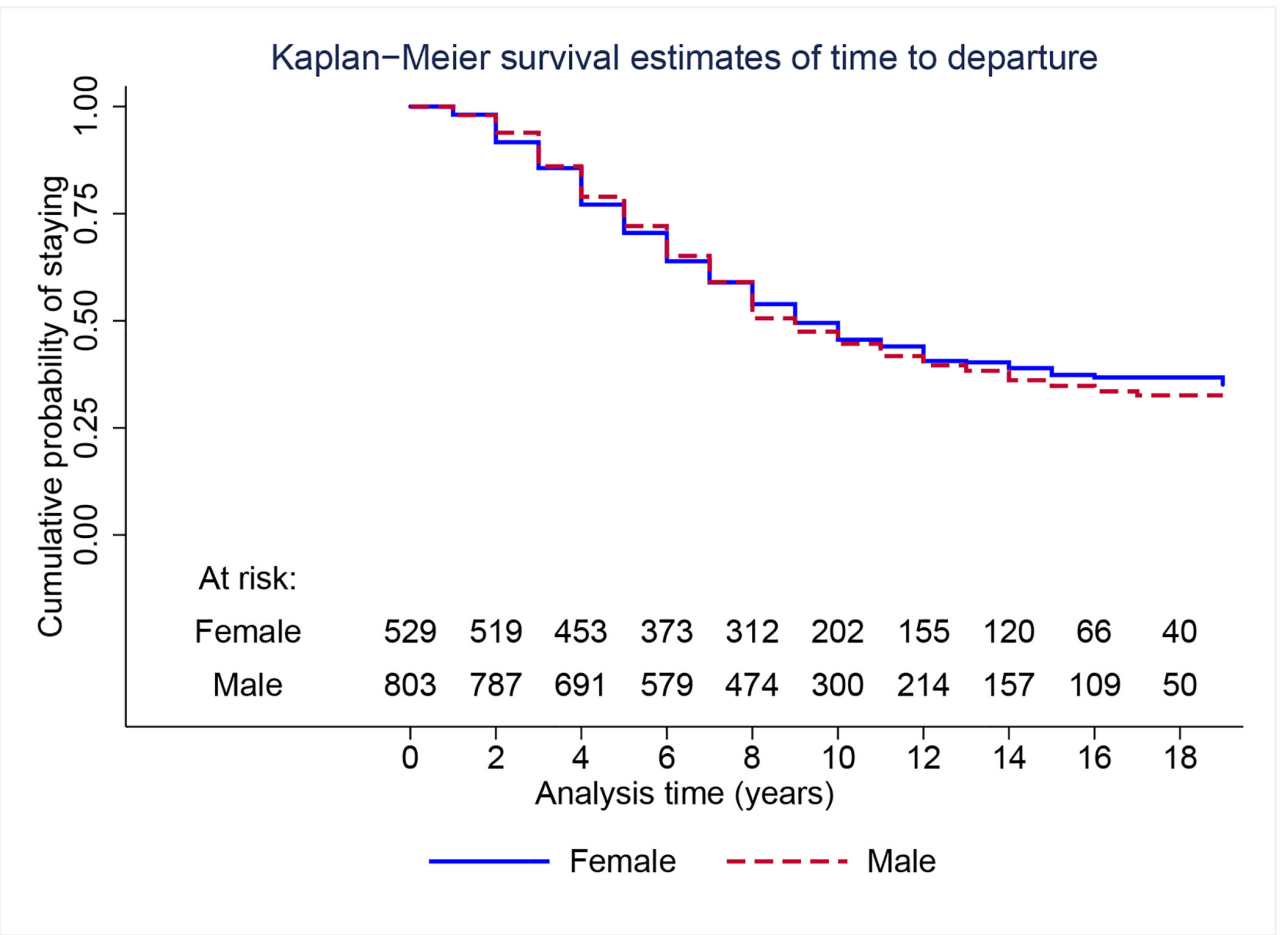
Nonparametric Kaplan-Meier survival curves of time to departure by gender for faculty who entered between 1990 and 2001 (cohorts 1–3).

We use survival analysis in order to determine if there are any systematic differences across genders in time of employment within the social sciences during the period covered by the study. Fig 1 shows the nonparametric Kaplan-Meier survival curve for the faculty that entered between 1990 and 2001 (cohorts 1–3) by gender. Visual inspection suggests no significant difference in departures rates between men and women. A log-rank test and a Wilcoxon test for the equality of the two survival curves yield *p*-values of 0.52 and 0.82, respectively, thus confirming that the two curves are statistically indistinguishable.

**Fig 2:**
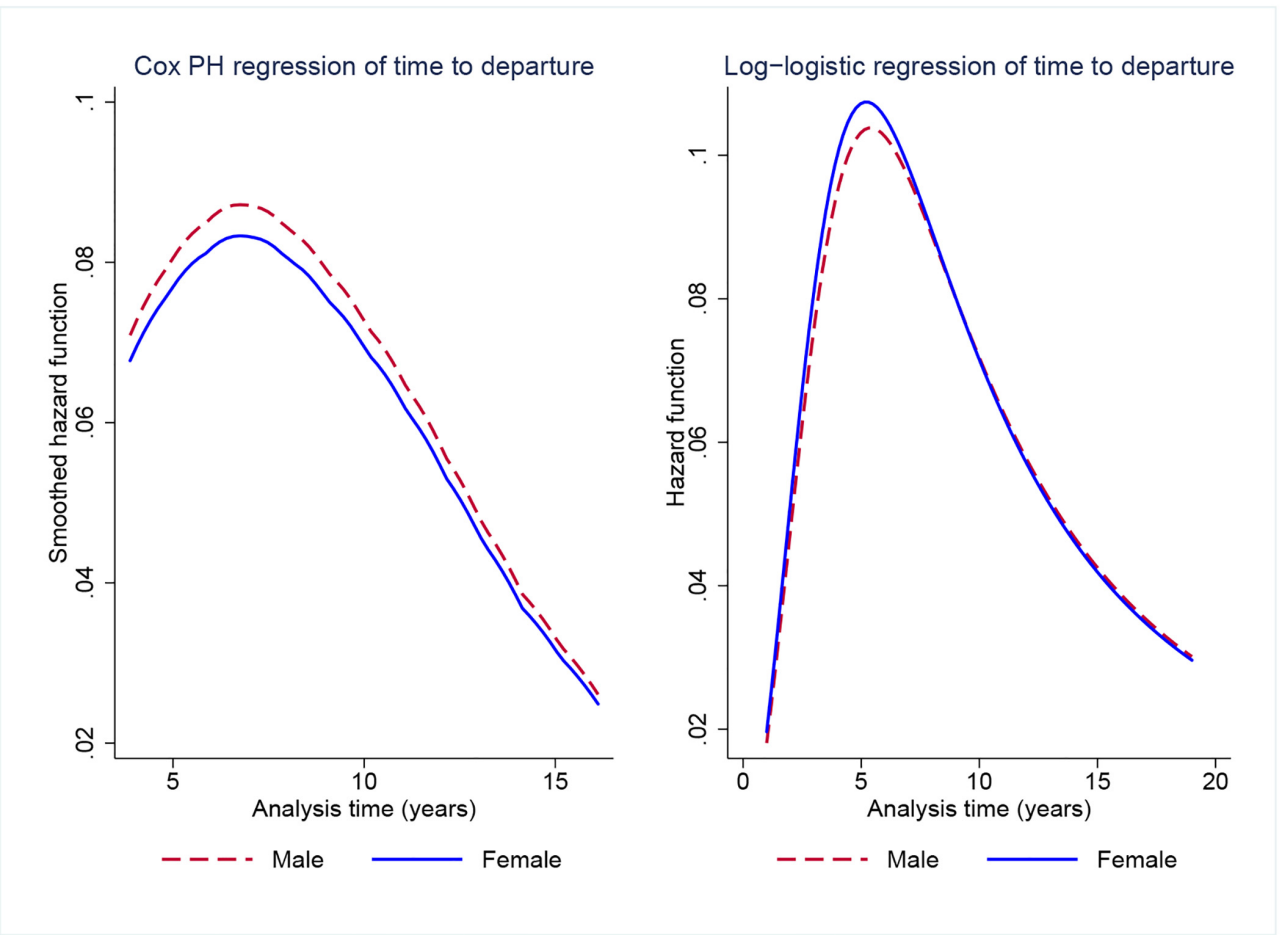
Survival analysis of time to departure for faculty who entered between 1990 and 2001 (cohorts 1–3) by gender. Estimates of the hazard function from Cox PH regression and log-logistic regression with gamma frailty.

Table 4 shows the estimation results for the semiparametric Cox regressions and the parametric frailty model. In model 1, a Cox model with the male indicator variable included on the right-hand side, the coefficient estimate for male gender is positive but statistically insignificant (*p* = 0.51). Model 2 in Table 4 is a parametric individual-frailty model used to account for unobserved heterogeneity. We tested different distributional specifications for the functional form of the baseline hazard: exponential, Weibull, log-normal, log-logistic, and Gompertz. We computed the AIC for each model to assess their fit, and the log-logistic specification, which is reported as model 2 in Table 4, provided the best fit for the data (lowest AIC). The log-logistic survival model with a gamma frailty also yields non-significant estimates of gender differences in departures rates. The coefficient on the male indicator variable is positive but statistically insignificant (*p* = 0.55). Note that a positive coefficient on the log-logistic regression has a different interpretation than a similarly signed coefficient in a Cox model. The log-logistic model is specified in accelerated failure time (AFT) form; that is, the natural logarithm of the survival time is expressed as a linear function of the covariates [47]. A positive coefficient in an AFT model expresses an increase in the expected waiting time to event, while a positive coefficient in the Cox PH model expresses a decrease in expected time to event or, equivalently, an increase in risk. In any case, estimates from both the Cox model (model 1) and the parametric frailty model (model 2) show no statistically discernible gender differences in time to departure. Moreover, the estimate for the frailty variance, *θ*, in model 2 is statistically significant. A likelihood-ratio test yields a chi-square statistic of 66.37 (*p* < 0.001), confirming that the frailty term is needed to model unobserved heterogeneity that would otherwise be unaccounted for. Fig 2 shows estimates of the hazard function for models 1 and 2 by gender. The function represents the instantaneous rate of failure, that is, the rate of departure at any given point in the academic career.

**Table 4:** Survival analysis of time to departure by gender (cohorts 1–3).

*Variable*	*(1)*		*(2)*		*(3)*		*(4)*		*(5)*	
	*Cox PH*		*Log-logistic model w/ gamma frailty*		*Cox PH (discipline-specific effects)*		*Log-logistic model w/ gamma frailty (discipline-specific effects)*		*Cox PH (cohort-specific effects)*	
	Coef.	*P > |z|*	Coef.	*P > |z|*	Coef.	*P > |z|*	Coef.	*P > |z|*	Coef.	*P > |z|*
	(s.e.)		(s.e.)		(s.e.)		(s.e.)		(s.e.)	
Male	.048	0.51	.034	0.55	-	-	-	-	-	-
	(.073)		(.057)							
Constant	-	-	1.931	0.00	-	-	2.038	0.00	-	-
			(.054)				(.107)			
Ln(gamma)	-	-	-.916	0.00	-	-	-.850	0.00	-	-
			(.048)				(.053)			
*Disciplines*										
Anth	-	-	-	-	-.879	0.00	.498	0.02	-	-
					(.261)		(.209)			
Comm	-	-	-	-	.162	0.39	-.190	0.22	-	-
					(.190)		(.154)			
Econ	-	-	-	-	.526	0.01	-.225	0.13	-	-
					(.192)		(.150)			
Geo	-	-	-	-	-.037	0.90	-.168	0.47	-	-
					(.308)		(.235)			
Poli	-	-	-	-	-.181	0.36	.044	0.77	-	-
					(.199)		(.154)			
Psy	-	-	-	-	-.151	0.40	-.011	0.93	-	-
					(.181)		(.142)			
Soc	-	-	-	-	-	-	-	-	-	-
Male × Anth	-	-	-	-	.479	0.11	-.208	0.36	-	-
					(.297)		(.229)			
Male × Comm	-	-	-	-	.027	0.88	-.046	0.76	-	-
					(.185)		(.154)			
Male × Econ	-	-	-	-	-.165	0.30	.084	0.49	-	-
					(.158)		(.124)			
Male × Geo	-	-	-	-	.033	0.92	.013	0.96	-	-
					(.344)		(.269)			
Male × Poli	-	-	-	-	-.035	0.85	.059	0.67	-	-
					(.181)		(.140)			
Male × Psy	-	-	-	-	-.051	0.75	.065	0.62	-	-
					(.165)		(.131)			
Male × Soc	-	-	-	-	-.237	0.23	.149	0.31	-	-
					(.198)		(.149)			
*Cohorts*										
Cohort 1	-	-	-	-	-	-	-	-	-.059	0.68
									(.143)	
Cohort 2	-	-	-	-	-	-	-	-	.037	0.79
									(.142)	
Cohort 3	-	-	-	-	-	-	-	-	-	-
Male × Cohort 1	-	-	-	-	-	-	-	-	.092	0.43
									(.119)	
Male × Cohort 2	-	-	-	-	-	-	-	-	.089	0.48
									(.127)	
Male × Cohort 3	-	-	-	-	-	-	-	-	-.030	0.82
									(.133)	
*Frailty term*										
Theta	-		1.194		-		.870		-	
			(.163)				(.173)			
LR χ^2^ (θ = 0)	-		66.27		-		30.24		-	
P > χ^2^	-		0.00		-		0.00		-	
N subjects	1332		1332		1332		1332		1332	
N failures	799		799		799		799		799	
Log-likelihood	-5371		-1511		-5334		-1497		-5370	
LR χ^2^	0.44		0.35		73.39		29.35		2.74	
P > χ^2^	0.50		0.55		0.00		0.01		0.73	
AIC	10744		3031		10695		3026		10750	

*Note*: Discipline abbreviations: Anth (Anthropology), Comm (Communication), Econ (Economics), Geo (Geography), Poli (Political Science), Psy (Psychology), Soc (Sociology).

Model 3 in Table 4 tests for discipline-specific gender patterns by breaking down the survival analysis of faculty departure by field. We show estimates of a Cox model where the male indicator variable is interacted with discipline dummy variables. Estimates of discipline-specific gender differences vary in sign, but none of the coefficients on the interaction terms are statistically significant. A standard Wald-type chi-square test does not reject the null hypothesis of joint equality of the coefficients (*p* = 0.56). In model 4, we test again for discipline-specific effects using the log-logistic survival model with a gamma frailty. We find no statistically significant gender differences. In other words, the absence of gender differences in the pooled sample seems not to be an artifact of aggregation, but a reflection of the absence of imbalances in each of the studied disciplines.

Additionally, we test for the possibility of heterogeneity in gender differences across different cohorts by interacting the male indicator variable with cohort dummies. Once again, none of the coefficients on the interactions are statistically significant, and the Wald-type chi-square test cannot reject the null hypothesis of joint equality of the coefficients (*p* = 0.76).

**Fig 3:**
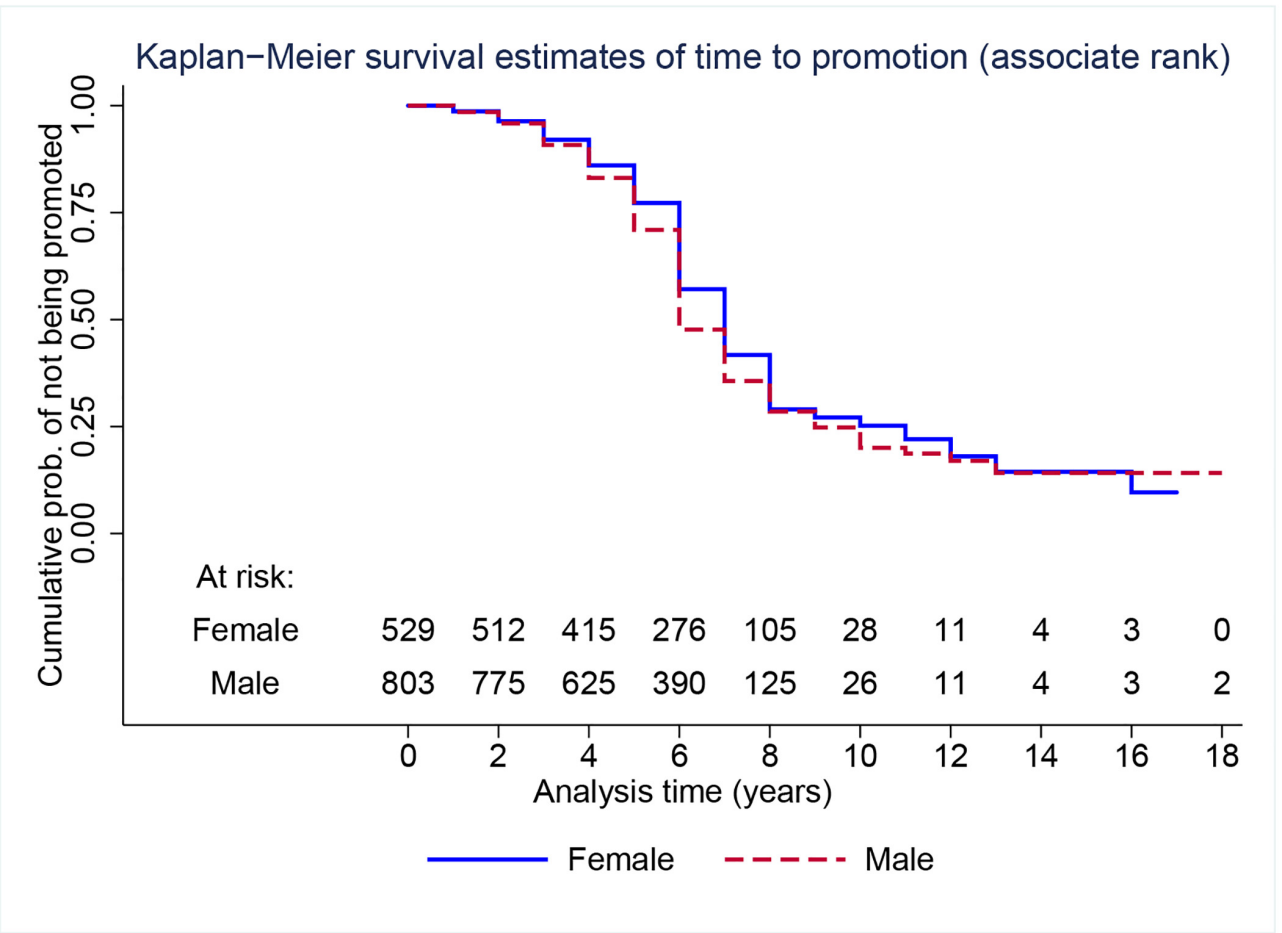
Nonparametric Kaplan-Meier survival curves of time to promotion to associate rank by gender for faculty who entered between 1990 and 2001 (cohorts 1–3).

We turn to the analysis of promotion from the assistant to the associate professor rank. Fig 3 shows Kaplan-Meier survival curves for the faculty that entered between 1990 and 2001 (cohorts 1–3) by gender. The survival curves suggest that male faculty are promoted at higher rates than female faculty. A log-rank test and a Wilcoxon test for the equality of the two survival curves yield *p*-values of 0.05 and 0.02, respectively, which suggests that men indeed experience lower time to tenure promotion than women.

**Fig 4:**
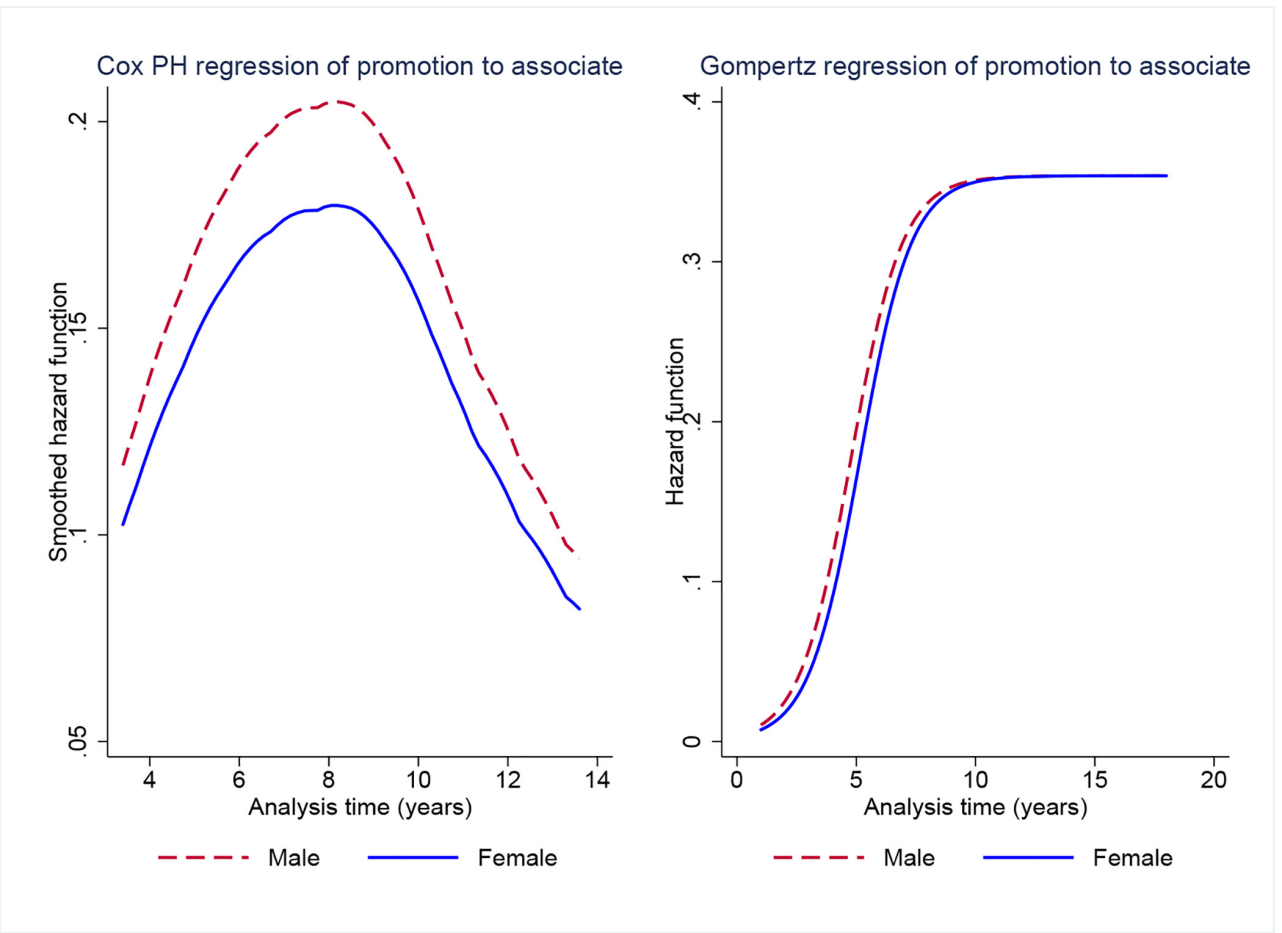
Survival analysis of time to promotion to associate rank for faculty who entered between 1990 and 2001 (cohorts 1–3) by gender. Estimates of the hazard function from Cox PH regression and Gompertz regression with gamma frailty.

As we did with the time-to-departure data, we employ Cox models and parametric frailty models to examine gender differences in promotion rates. Table 5 shows the results of these estimations. The coefficient estimate from a Cox model including a male indicator variable only (model 6) is positive, but falls short of conventional levels of statistical significance (*p* = 0.06). We again test several parametric specifications for the individual-frailty model and, based on the criterion of lowest AIC, we report estimates from a Gompertz specification (model 7). We find a positive coefficient on the male indicator and the estimate is statistically significant (*p* = 0.02). The Gompertz model is specified in log relative-hazard form, so a positive coefficient on the male variable indicates that time to promotion is lower for men than for women. The estimate of the frailty variance, *θ*, in model 7 is also statistically significant (*p* < 0.001), suggesting that there is indeed unobserved heterogeneity in time to promotion that is accounted for by the frailty term. Fig 4 shows estimates of the hazard function for models 6 and 7 by gender.

**Table 5:** Survival analysis of time to promotion from assistant to associate rank by gender (cohorts 1–3).

*Variable*	*(6)*		*(7)*		*(8)*		*(9)*		*(10)*	
	*Cox PH*		*Gompertz model w/ gamma frailty*		*Cox PH (discipline-specific effects)*		*Gompertz model w/ gamma frailty (discipline-specific effects)*		*Cox PH (cohort-specific effects)*	
	Coef.	*P > |z|*	Coef.	*P > |z|*	Coef.	*P > |z|*	Coef.	*P > |z|*	Coef.	*P > |z|*
	(s.e.)		(s.e.)		(s.e.)		(s.e.)		(s.e.)	
Male	.150	0.06	.348	0.02	-	-	-	-	-	-
	(.078)		(.154)							
Constant	-	-	-5.836	0.00	-	-	-6.235	0.00	-	-
			(.232)				(.376)			
Gamma	-	-	.928	0.00	-	-	.939	0.00	-	-
			(.056)				(.058)			
*Disciplines*										
Anth	-	-	-	-	.464	0.04	.578	0.20	-	-
					(.221)		(.455)			
Comm	-	-	-	-	.173	0.45	.377	0.39	-	-
					(.229)		(.444)			
Econ	-	-	-	-	-.499	0.09	-.489	0.35	-	-
					(.295)		(.528)			
Geo	-	-	-	-	.402	0.22	.702	0.29	-	-
					(.329)		(.667)			
Poli	-	-	-	-	.341	0.11	.320	0.45	-	-
					(.216)		(.422)			
Psy	-	-	-	-	.525	0.01	.643	0.10	-	-
					(.197)		(.389)			
Soc	-	-	-	-	-	-	-	-	-	-
Male × Anth	-	-	-	-	.260	0.25	1.097	0.03	-	-
					(.227)		(.498)			
Male × Comm	-	-	-	-	.151	0.51	.140	0.74	-	-
					(.228)		(.428)			
Male × Econ	-	-	-	-	.604	0.03	.841	0.08	-	-
					(.272)		(.472)			
Male × Geo	-	-	-	-	.236	0.51	1.008	0.17	-	-
					(.356)		(.742)			
Male × Poli	-	-	-	-	.099	0.58	.388	0.28	-	-
					(.179)		(.360)			
Male × Psy	-	-	-	-	-.110	0.49	-.213	0.50	-	-
					(.160)		(.319)			
Male × Soc	-	-	-	-	.631	0.00	.975	0.02	-	-
					(.208)		(.404)			
*Cohorts*										
Cohort 1	-	-	-	-	-	-	-	-	-.096	0.52
									(.150)	
Cohort 2	-	-	-	-	-	-	-	-	-.006	0.96
									(.150)	
Cohort 3	-	-	-	-	-	-	-	-	-	-
Male × Cohort 1	-	-	-	-	-	-	-	-	.224	0.09
									(.132)	
Male × Cohort 2	-	-	-	-	-	-	-	-	.186	0.20
									(.144)	
Male × Cohort 3	-	-	-	-	-	-	-	-	.057	0.67
									(.133)	
*Frailty term*										
Theta	-		2.625		-		2.614		-	
			(.295)				(.301)			
LR χ^2^ (θ = 0)	-		301		-		289		-	
P > χ^2^	-		0.00		-		0.00		-	
N subjects	1332		1332		1332		1332		1332	
N failures	684		684		684		684		684	
Log-likelihood	-4326		-811		-4307		-798		-4325	
LR χ^2^	3.70		5.08		41.54		31.75		5.20	
P > χ^2^	0.05		0.02		0.00		0.00		0.39	
AIC	8655		1631		8641		1628		8661	

*Note*: Discipline abbreviations: Anth (Anthropology), Comm (Communication), Econ (Economics), Geo (Geography), Poli (Political Science), Psy (Psychology), Soc (Sociology).

We test for discipline-specific gender patterns in time to promotion to the associate rank. Table 5 reports estimates of a Cox model where the male indicator variable is interacted with discipline dummy variables (model 8). With the exception of psychology, all coefficients on the discipline-specific male indicators are positive. Only the coefficient estimates for economics and sociology are statistically significant (*p* = 0.03 and *p* = 0.003, respectively). A Wald-type chi-square test, however, fails to reject the null hypothesis of joint equality of the coefficients on the interaction terms (*p* = 0.10). We also test for discipline-specific effects using a frailty model (model 9). Again, with the exception of psychology, all coefficients are positive. The estimates for anthropology and sociology are statistically significant (*p* = 0.03 and 0.02, respectively). A Wald-type chi-square test fails to reject the null hypothesis of joint equality of the coefficients on the interaction terms (*p* = 0.15). The Cox and the parametric frailty model agree on the significant effect for sociology. Additionally, in model 10 we test for the possibility of heterogeneity in gender differences across different cohorts. None of the coefficients on the interactions are statistically significant, and a chi-square test cannot reject the null hypothesis of joint equality of the coefficients (*p* = 0.19).

**Fig 5:**
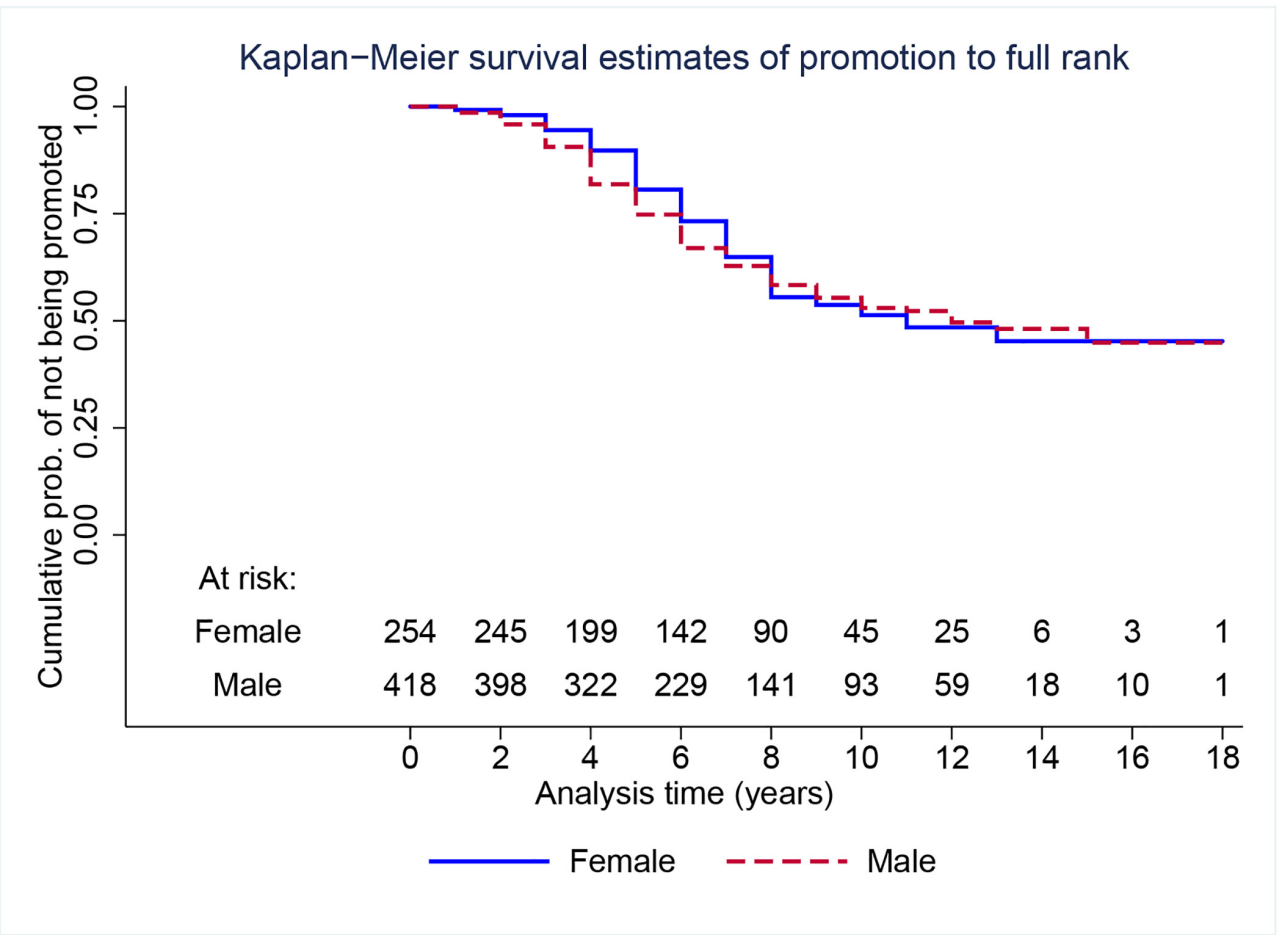
Nonparametric Kaplan-Meier survival curves of time to promotion to full rank by gender for faculty who entered between 1990 and 2001 (cohorts 1–3).

Finally, we present results of the analysis of time to promotion from associate to full professor by gender, conditional on the faculty member having been promoted to associate professor. Fig 5 shows Kaplan-Meier survival curves for the faculty that entered between 1990 and 2001 (cohorts 1–3) by gender. A log-rank test and a Wilcoxon test for the equality of the two curves yield *p*-values of 0.58 and 0.16, respectively, suggesting that the survival curves for men and women are not statistically distinguishable.

**Fig 6:**
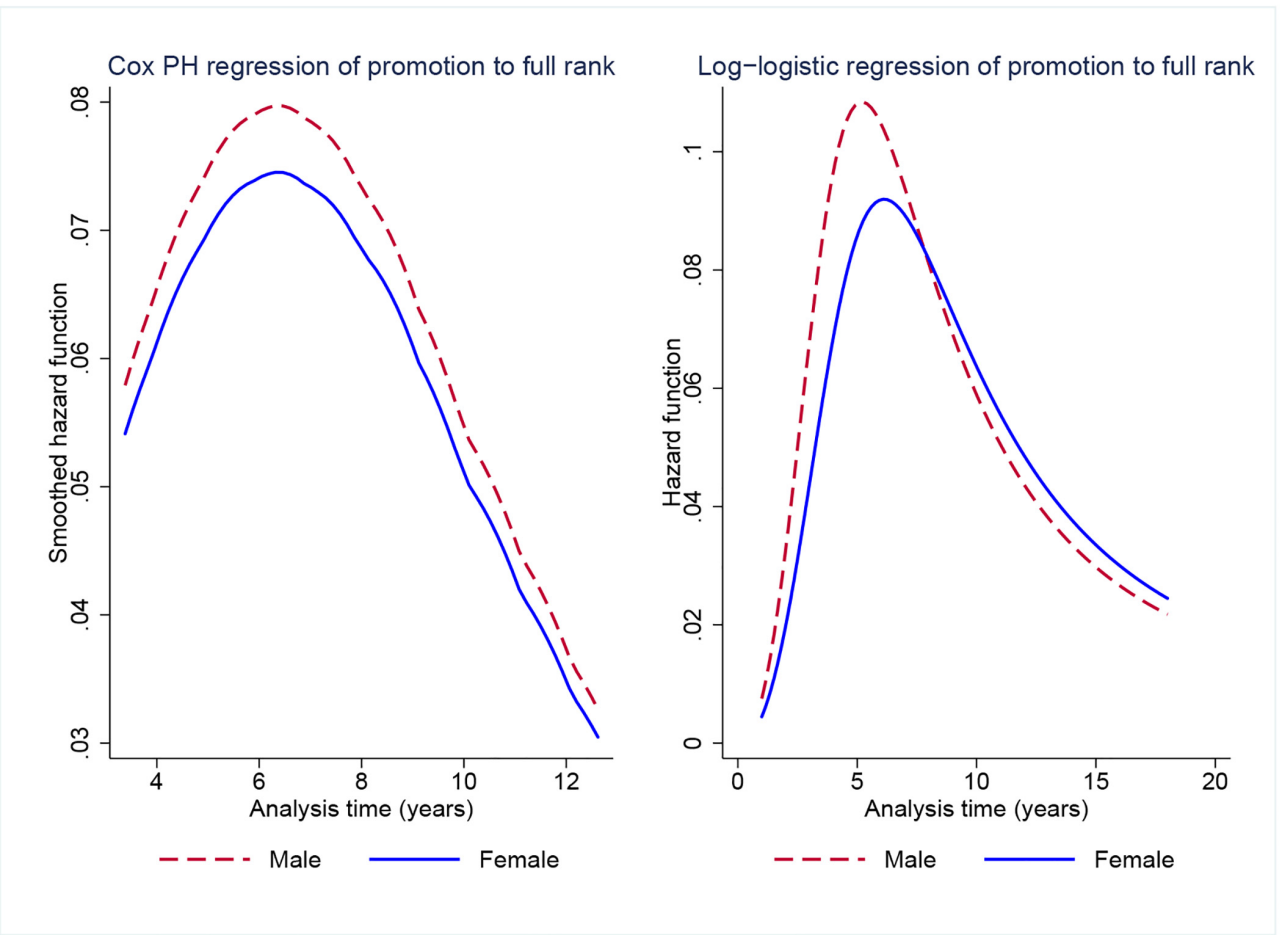
Survival analysis of time to promotion to full rank for faculty who entered between 1990 and 2001 (cohorts 1–3) by gender. Estimates of the hazard function from Cox PH regression and log-logistic regression with gamma frailty.

Table 6 shows estimates of Cox regressions and parametric frailty models. The estimate from a Cox model including a male indicator variable only (model 11) is positive but not statistically significant (*p* = 0.60). With the lowest AIC, the log-logistic parameterization of the baseline hazard for the individual-frailty model provides the best fit for the data (model 12). We find a negative and statistically significant (*p* = 0.03) coefficient on the male indicator variable, suggesting that time to promotion from associate to full rank is shorter for men than for women. Fig 6 shows estimates of the hazard function for models 11 and 12 by gender.

**Table 6:** Survival analysis of time to promotion from associate to full rank by gender (cohorts 1–3).

*Variable*	*(11)*		*(12)*		*(13)*		*(14)*		*(15)*	
	*Cox PH*		*Log-logistic model w/ gamma frailty*		*Cox PH (discipline-specific effects)*		*Log-logistic model w/ gamma frailty*		*Cox PH (cohort-specific effects)*	
							*(discipline-specific effects)*			
	Coef.	*P > |z|*	Coef.	*P > |z|*	Coef.	*P > |z|*	Coef.	*P > |z|*	Coef.	*P > |z|*
	(s.e.)		(s.e.)		(s.e.)		(s.e.)		(s.e.)	
Male	.070 (.134)	0.60	-.164 (.077)	0.03	-	-	-	-	-	-
Constant	-	-	2.051	0.00	-	-	1.904	0.00	-	-
			(.071)				(.154)			
Ln(gamma)	-	-	-1.164	0.00	-	-	-1.299	0.00	-	-
			(.084)				(.082)			
*Disciplines*										
Anth	-	-	-	-	-.154	0.69	.296	0.13	-	-
					(.393)		(.194)			
Comm	-	-	-	-	-.569	0.21	.382	0.08	-	-
					(.456)		(.219)			
Econ	-	-	-	-	.211	0.67	-.802	0.01	-	-
					(.500)		(.321)			
Geo	-	-	-	-	-.859	0.26	.513	0.13	-	-
					(.764)		(.338)			
Poli	-	-	-	-	.257	0.49	.005	0.98	-	-
					(.372)		(.189)			
Psy	-	-	-	-	.275	0.43	.049	0.78	-	-
					(.347)		(.177)			
Soc	-	-	-	-	-	-	-	-	-	-
Male × Anth	-	-	-	-	-.091	0.82	-.085	0.68	-	-
					(.403)		(.207)			
Male × Comm	-	-	-	-	-.662	0.25	.486	0.05	-	-
					(.570)		(.245)			
Male × Econ	-	-	-	-	.109	0.80	.399	0.18	-	-
					(.440)		(.301)			
Male × Geo	-	-	-	-	1.262	0.10	-.774	0.02	-	-
					(.768)		(.345)			
Male × Poli	-	-	-	-	-.245	0.40	.034	0.82	-	-
					(.291)		(.148)			
Male × Psy	-	-	-	-	.136	0.59	-.191	0.14	-	-
					(.254)		(.129)			
Male × Soc	-	-	-	-	-.013	0.97	.049	0.80	-	-
					(.372)		(.195)			
*Cohorts*										
Cohort 1	-	-	-	-	-	-	-	-	.052	0.87
									(.331)	
Cohort 2	-	-	-	-	-	-	-	-	-.099	0.77
									(.343)	
Cohort 3	-	-	-	-	-	-	-	-	-	-
Male × Cohort 1	-	-	-	-	-	-	-	-	-.050	0.79
									(.187)	
Male × Cohort 2	-	-	-	-	-	-	-	-	.091	0.70
									(.238)	
Male × Cohort 3	-	-	-	-	-	-	-	-	.345	0.31
									(.341)	
*Frailty term*										
Theta	-		2.110		-		2.258		-	
			(.432)				(.401)			
LR χ^2^ (θ = 0)	-		31.73		-		44.34		-	
P > χ^2^	-		0.00		-		0.00		-	
N subjects	672		672		672		672		672	
N failures	239		239		239		239		239	
Log-likelihood	-1408		-490		-1392		-462		-1406	
LR χ^2^	0.28		4.40		30.99		59.65		3.26	
P > χ^2^	0.60		0.04		0.00		0.00		0.66	
AIC	2818		988		2811		957		2823	

*Note*: Discipline abbreviations: Anth (Anthropology), Comm (Communication), Econ (Economics), Geo (Geography), Poli (Political Science), Psy (Psychology), Soc (Sociology).

We test for discipline-specific gender patterns in time to promotion to full professor. Estimates of a Cox model where the male indicator variable is interacted with discipline dummy variables are reported as model 13. We find no particular evidence of discipline-specific gender differences, as all coefficients on the interaction terms are statistically insignificant. A chi-square test of the joint equality of the coefficients on the interaction terms fails to reject the null (*p* = 0.52). Alternatively, a frailty model with a log-logistic parameterization for the baseline hazard (model 14) yields statistically significant estimates for the male indicator variable for the disciplines of communication and geography. The coefficient for communication is positive, which means that the time to promotion from associate to full rank is on average greater for men than for women in that discipline. The coefficient for the field of geography, on the other hand, is negative, indicating that the time to promotion to full professor rank in that discipline is greater for women than for men. A chi-square test of the joint equality of the coefficients on the interaction terms rejects the null at the conventional level of significance (*p* = 0.04). Moreover, a test for the possibility of heterogeneity in gender differences in time to promotion to full rank across different cohorts (model 15) yields no significant results.

## Discussion and Conclusion

Taken together, our results provide mixed evidence of significant differences in time to departure and time to promotion by gender. While we cannot discern any differences between men and women with respect to retention rates, we do find evidence that men have a higher chance of being promoted from assistant to associate professor. Furthermore, in the breakdown by discipline, the evidence points consistently towards higher rates of promotion for men than for women in sociology and, to a lesser degree, economics. When it comes to promotion to full professor, the evidence is inconclusive. We find significant gender differences in some model specifications, but overall the effect is sensitive to changes in specification. Moreover, the direction of the effect does not always point unambiguously towards a disadvantage for women. When we break down the analysis by discipline, men seem to have a higher chance of promotion to full professor in geography, but the opposite seems to be true for communication.

Our results corroborate and contribute to the generalizability of the findings on faculty retention in Kaminski and Geisler [2]. These authors find no clear evidence of differential departure rates by gender in the sciences and engineering and we find similar results for the social sciences. Moreover, by adding a frailty model to the analysis, which accounts for unobserved heterogeneity in faculty retention, we lend further credibility to this finding. Consistent with Wolfinger et al. [15], Long et al. [22], and Junn [27], we find significant gender differences when it comes to promotion to the associate level. We find that women are systematically less likely to be promoted from assistant to associate professor. On the other hand, while Long et al. and Wolfinger et al. find significant gender differences in promotion to full professor, our results are less conclusive.

On balance, the analysis hints at some positive developments in gender inequality in academic careers. Although the temporal coverage of the data is too short to detect a clear trend, there seems to have been an overall increase in the percentage of women hired in the social sciences since 1990, as evidenced in Table 1. Moreover, a comparison with the numbers presented in Kaminski and Geisler indicate that the proportion of women hired appears to be substantially larger in the social sciences than in the STEM disciplines [2]. While the figures presented here still suggest a situation of less than parity in hiring, our findings seem to corroborate the claim that the hiring and retention trends by gender “imply an increased presence of women in [the analyzed] departments over the long run” [2]. Persistent differences in tenure promotion, however, give us reason to be cautious in extrapolating any future advances.

Our findings provide only an incipient picture of gender inequalities when it comes to faculty retention in American research institutions. Existing studies—this one included—examine faculty departure in general, but overlook important differences in the reasons for departure. In the existing data, every event of departure contributes the same information to the analysis. However, there is reason to believe that gender inequality in faculty retention might manifest itself in different ways depending on the reasons that lead faculty members to leave their institutions. Some leave for personal and family reasons, some leave because of an unsupportive work environment, others leave in anticipation of or as the result of a negative tenure decision, and other still leave for a better position elsewhere [42]. Gender dynamics should play a different role in each of these decisions to leave, such that differences in departure rates between men and women might exist in some cases but not others. Inequality in departure rates caused by differential rates of tenure denial, for example, would have completely different implications for gender relations than inequality in the acceptance of better positions.

In other words, all departures are not equal, and the various motivations for the decision to leave contribute important information and should be incorporated into studies of faculty retention. The way departure events are coded in the existing data, therefore, fails to take into account all the relevant information involved in a faculty’s motivation to leave, thus obscuring important nuances in the phenomenon of faculty retention and limiting the inferences that can be made from the data. The disaggregation of departure events into multiple types of events is a necessary next step in the study of faculty retention. Future research would benefit from universities sharing data that detail the different circumstances that lead researchers to depart, such as tenure denial data. This would involve access to the records of institutions along the lines of the study conducted by Welch and Long [49]. Such data could be analyzed under a “competing risks” framework, in which gender disparities in faculty retention are allowed to vary across different types of risks, that is, different types of departure events. This design would allow for a more thorough and informative examination of gender inequalities in faculty retention and promotion in U.S. research institutions.

It would also be opportune to include data on race and ethnicity in future analyses—following Junn [27]—so that racial inequalities in faculty retention may also be further investigated. Cooperation from research universities in sharing data would be particularly useful, given that data on racial and ethnic attributes are not readily available and are often incomplete in public sources. Purchasing these data from marketing firms would be prohibitively expensive.

Further study of the processes and mechanisms that bring about the underrepresentation of women in research careers is a worthwhile effort. Here we took another step in that direction and proposed a few more. As emphasized by the National Academy of Sciences et al., “women who are interested in science and engineering careers are lost at every educational transition” [3]. Important policies have been put in place in an attempt to correct imbalances, and progress has been slow but steady. Further engagement from university faculty, administrators, and funding agencies will be crucial for the continued advancement of policies and rigorous assessment of gender-based trends in the academic profession.

## Supporting Information

S1 Data (DOCX)
